# Cross-disorder and disorder-specific deficits in social functioning among schizophrenia and alzheimer’s disease patients

**DOI:** 10.1371/journal.pone.0263769

**Published:** 2022-04-14

**Authors:** Ilja M. J. Saris, Moji Aghajani, Niels Jongs, Lianne M. Reus, Nic J. A. van der Wee, Amy C. Bilderbeck, Inge Winter van Rossum, Celso Arango, Alejandro de la Torre-Luque, Asad Malik, Andreea Raslescu, Gerard R. Dawson, José L. Ayuso-Mateos, Martien J. Kas, Brenda W. J. H. Penninx

**Affiliations:** 1 Department of Psychiatry, Amsterdam Neuroscience and Amsterdam Public Health Research Institute, Amsterdam UMC, Vrije Universiteit and GGZ inGeest, Amsterdam, the Netherlands; 2 Institute of Education & Child Studies, Section Forensic Family & Youth Care, Leiden University, Leiden, the Netherlands; 3 Groningen Institute for Evolutionary Life Sciences, University of Groningen, Groningen, the Netherlands; 4 Department of Neurology, Alzheimer Center Amsterdam, Amsterdam Neuroscience, Vrije Universiteit Amsterdam, Amsterdam UMC, Amsterdam, the Netherlands; 5 Department of Psychiatry, Leiden University Medical Centre, Leiden, the Netherlands; 6 Leiden Institute for Brain and Cognition, Leiden, the Netherlands; 7 P1vital Ltd., Wallingford, Oxfordshire, United Kingdom; 8 Department of Psychiatry, University Medical Center Utrecht Brain Center, Utrecht University, Utrecht, the Netherlands; 9 Hospital General Universitario Gregorio Marañón, CIBERSAM, IiSGM, Universidad Complutense, School of Medicine, Madrid, Spain; 10 Centre of Biomedical Research in Mental Health (CIBERSAM), Barcelona, Spain; 11 Universidad Complutense de Madrid, Madrid, Spain; 12 Department of Psychiatry, Hospital Universitario la Princesa (IIS-Princesa), Universidad Autonoma de Madrid, Madrid, Spain; Universidad Nacional Autonoma de Mexico, MEXICO

## Abstract

**Background:**

Social functioning is often impaired in schizophrenia (SZ) and Alzheimer’s disease (AD). However, commonalities and differences in social dysfunction among these patient groups remain elusive.

**Materials and methods:**

Using data from the PRISM study, behavioral (all subscales and total score of the Social Functioning Scale) and affective (perceived social disability and loneliness) indicators of social functioning were measured in patients with SZ (*N* = 56), probable AD (*N* = 50) and age-matched healthy controls groups (HC, *N* = 29 and N = 28). We examined to what extent social functioning differed between disease and age-matched HC groups, as well as between patient groups. Furthermore, we examined how severity of disease and mood were correlated with social functioning, irrespective of diagnosis.

**Results:**

As compared to HC, both behavioral and affective social functioning seemed impaired in SZ patients (Cohen’s *d’s* 0.81–1.69), whereas AD patients mainly showed impaired behavioral social function (Cohen’s *d’s* 0.65–1.14). While behavioral indices of social functioning were similar across patient groups, SZ patients reported more perceived social disability than AD patients (Cohen’s *d’s* 0.65). Across patient groups, positive mood, lower depression and anxiety levels were strong determinants of better social functioning (p’s <0.001), even more so than severity of disease.

**Conclusions:**

AD and SZ patients both exhibit poor social functioning in comparison to age- and sex matched HC participants. Social dysfunction in SZ patients may be more severe than in AD patients, though this may be due to underreporting by AD patients. Across patients, social functioning appeared as more influenced by mood states than by severity of disease.

## Introduction

Social functioning is crucial for human survival and it has been established that ‘loneliness kills’ [1, 2]. Social functioning entails a collection of intricate and multi-factorial behavioral repertoires, which seem to be facilitated by many complex brain network processes [3–5]. Social dysfunction could be broadly defined as inability of the individual to “*integrate behavioral*, *cognitive and affective skills to flexibly adapt to diverse social context and demands”* [6]. Because of its complexity, social dysfunction often arises as one of the first symptoms in neuropsychiatric disorders such as Schizophrenia (SZ) and Alzheimer’s disease (AD) [3]. However, the origins of social dysfunction in these disorders remain poorly understood [7, 8]. Specifically, while SZ and AD patients are characterized by distinctive psychopathologies, their psychosocial deficits are deemed partly overlapping. However, empirical data on behavioral and neurobiological commonalities and differences is practically lacking.

The definition of ‘social dysfunction’ has specific focal points dependent on the disorder being studied, with a broad range in study methods and outcome measures as a consequence. The neurobiological underpinnings of the subjective, affective evaluation of social interactions (i.e. loneliness, self-perceived social capacities) are thought to differ from the ones underlying the more objective, behavioral aspects of social interactions (i.e. frequency of participating in social activities, hours spend alone) [4, 9, 10]. We therefore differentiate between behavioral and affective indicators of social functioning, in line with prior work by our group and other studies [9, 11–13]. Social dysfunction in SZ is described in a large and growing body of literature that has identified severe impairments in mentalizing, interpersonal interaction, regulating emotions and emotion decoding in social situations [14–17]. It has been argued that higher order affective social processes such as mentalizing are more prone to deficits in SZ patients than processes which require less mental effort, such as emotion recognition and mirroring [14, 18]. In addition, the behavioral pattern of social withdrawal is described as a prodromal symptom of SZ, with an onset up to several years before the first psychotic episode [19]. In contrast to SZ, empirical data on social dysfunction in AD is arguably more sparse. Social dysfunction in AD typically starts with initially subtle impairments in social and affective cognition, which worsen as the disorder progresses [20, 21]. Social impairment has been described as a distinct constellation of AD symptoms, possibly explained by the degenerative processes differentially affecting brain regions [22]. More specifically, in AD social dysfunction seems to be associated with impairments in interpreting cues to others’ emotional states, and thus identifying others’ emotions, i.e. affective aspects of social dysfunction [21]. In addition, it has been described that the judgments of AD patients about their own social functioning is more positive than according to the perception of the caregiver [20, 23, 24]. In sum, social functioning in both SZ and AD seems most affected in understanding others [8].

The notion of highly overlapping social functioning deficits in neuropsychiatric disorders has gained more support and interest in recent years, especially since the launch of the RDoC framework and the EU-ROAMER initiatives, which advocate a deeper understanding of trans-diagnostic clinical phenomena and their neurobiobehavioral underpinnings [25, 26]. Currently, a clear understanding of what transdiagnostic research exactly entails, is still under debate and clear guidelines are lacking [27, 28]. One of the proposed, stringent guidelines for transdiagnostic research are the four Mansell criteria: (1) It describes both a clinical, and (2) a non-clinical sample, (3) it must be present in at least four disorders and (4) the transdiagnostic construct must be demonstrated in all mental disorders investigated [29]. However, before being able to examine such underpinnings, it is necessary to more fully grasp the differences and commonalities in a complex phenomenon such as social dysfunction–including both behavioral and affective aspects—across neuropsychiatric disorders.

OeHoThe OeHomain aim of the present study is to examine differences and commonalities in affective and behavioral indicators of social functioning among SZ and AD patients, as compared to age and sex-matched healthy controls (HC), as well as compared to each other (SZ versus AD). Ultimately this could guide future neurobiological research on the transdiagnostic domain of social functioning, however, not fulfilling the Mansell criteria in this study, we will use cross-disorder instead as preferred terminology. The current study is part of the larger EU-funded PRISM Project (Psychiatric Ratings using Intermediate Stratified Markers), which examines the neurobiobehavioral underpinnings of social dysfunction in order to advance and formulate more effective treatment strategies [30]. Whereas SZ and AD differ in core symptoms, genetic profile, obvious age difference and underlying neurobiology, they importantly seem to overlap considerable in social functioning deficits (i.e., social withdrawal, interpersonal functioning, loneliness) [30, 31]. A uniform assessment of social functioning is essential to progress the field of transdiagnostic analyses. Studying patients with distinctively different neuropsychiatric disorders also aligns nicely with the RDoC perspective that clinical psychological problems are best defined along functional domains with shared neurobiological substrates, regardless of diagnostic nosologies, to attain novel insights and advance treatment [25]. We aimed to include patients with a relatively recent disease onset to capture as much as possible the underlying neurobiology of *social dysfunction* rather than long-term consequences of psychopathology or neurodegeneration. A transdiagnostic approach of investigating social (dys)function with these two discrete clinical entities may thus elucidate both disorder-specific and cross-disorder deficits.

## Material and methods

### Participants

Data for the current study were derived from the PRISM study, which examines social dysfunction as a transdiagnostic symptom in individuals with SZ (N = 56), probable AD (N = 52), and age-matched HC participants (two groups, N = 29 for younger HC (age 18–45) and N = 28 for older HC (age 50–80) [30]. Two participants (AD patients) did not complete social functioning questionnaires, leaving 163 participants for analyses.

Participants were recruited between July 2017 and March 2019 from five different recruiting sites across Spain (Hospital General Universitario Gregorio Marañón and Hospital Universitario de La Princesa) and the Netherlands (University Medical Center Utrecht, VU University Medical Center Amsterdam and Leiden University Medical Center). The study was approved by the Ethics Review Board of all participating centers: University Medical Center Utrecht, VU University Medical Center Amsterdam, Leiden University Medical Center, Hospital General Universitario Gregorio Marañón and Hospital Universitario de La Princesa. All participants provided verbal and written informed consent. Rationale and clinical implementation for the PRISM study is described in depth elsewhere [4, 30, 32].

### In- and exclusion criteria

SZ patients were eligible if they had a) a diagnosis of schizophrenia (confirmed using DSM-based Mini-International Neuropsychiatric Interview (MINI) assessment), b) had a maximum of 15-year disease duration since diagnosis, c) an age between 18–45 years, and d) a score of ≤22 on the 7-item positive subscale of the positive and negative syndrome scale (PANSS) [33] to rule out an active psychotic episode hampering adequate study participation [32]. SZ patients were excluded when they were, in the clinician’s judgment, a danger to themselves or others. AD patients were eligible if they had: a) a diagnosis of probable AD (meeting the National Institute on Aging and the Alzheimer’s Association criteria), b) a Mini-Mental State Examination (MMSE) [34] score between 20–26 (indicating mild AD pathology), c) an aged between 50–80 years. Multiple strokes, either based on clinical judgement, medical history or imaging results were exclusion criteria for the AD patient group.

Because AD is a progressive disorder with–after diagnosis- a quite variable disease course, we have included AD patients based on their cognitive abilities as measured by the MMSE [34]. Since social impairments may also stem from altered social interactions during ‘sick years’, SZ patients were included with a maximum of 15-year disease duration since diagnosis.

For both the SZ and AD patient groups, we had additional exclusion criteria: a) diagnosis of a severe, current Major Depressive Disorder (MDD) DSM-IV diagnosis (as assessed with the MINI) [35] and with a Quick Inventory of Depressive Symptomatology, Self-Rated (QIDS-SR) [36] ≥16), b) diagnosis of any other *primary* psychiatric diagnosis that requires intervention; c) alcohol or drug abuse/dependence within previous 3 years (as assessed on the MINI), d) severe Parkinsonism as a consequence of antipsychotic medication (as assessed with a score ≥4 on the Extrapyramidal Symptom Rating Scale) [37], e) unstable comorbid somatic disorders potentially affecting the central nervous system (CNS), f) unstable use of medication that could affect CNS (e.g. start of or changed dosage within last 8 weeks).

We included two HC groups, matching on sex and age with the SZ (between 18–45 years) and AD (between 50–80 years) groups. Scores on the MMSE for the older HC participants should be comparable to normative data according to age and years of education. Exclusion criteria for the HC groups were: a) history of psychiatric Axis-I disorder (as confirmed by the MINI) or neurological disease associated with cognitive impairment; b) mild or more severe depression (score >5 on the QIDS-SR); c) current or prior use of antidepressant or anxiolytic medication including benzodiazepines, or other prescribed medication in the last 6 weeks that may affect the CNS.

### Behavioral and affective social functioning indicators

Behavioral indicators of social functioning—The Social Functioning Scale (SFS) consists of seven subscales; social withdrawal, interpersonal functioning, competence and performance independence, recreational and prosocial activities, and employment. The subscale ‘employment’ was not used for total scale analyses, since most participants were retired in the older HC and AD group introducing a bias as confirmed by the significant association with age and in line with reporting in a previous study [38]. We conducted Principal Component Analyses with oblique rotation as the variables are correlated to test if the different subscales represent one factor (i.e. behavioral social functioning) or two (i.e. affective and behavioral aspects of social functioning). In line with other studies using the SFS [38–40], the factor analysis confirmed that the six SFS subscales reflect one component (see S1 Table). The social withdrawal subscale (higher score indicates less social withdrawal) focuses on time spend alone. The interpersonal functioning subscale includes ability to have rational conversation and difficulty talking to people. The subscale independence-competence and independence-performance are two identical lists of activities (e.g. buying items from shops alone) where participants judge whether they *think* they are able to do these independently (competence) and following what they actually independently *did* in the past three months (performance). The subscales, recreational and pro-social activities, consist of a list with several activities such as visiting relatives or playing sports, and the frequency in which participants engage in that activity. We have followed the original scoring guidelines [39], which advocate using Social Functioning Scale total scores (Cronbach’s alpha 0.80), as these better captures underlying social functions/behaviors, and also safeguards reproducibility across studies. However, since the different subscales might reflect differences in impairments, we opted to also show the various subscales in subsequent supplementary analyses (S2 Table).

Affective indicators of social functioning—Loneliness was assessed with the 11-item de Jong Gierveld loneliness scale [41], which examines feelings of loneliness (Cronbach’s alpha 0.8). Perceived social disability was measured with an adjusted 5-item ‘getting along’ subdomain from the WHODAS 2.0 (WHODAS 2.0: World Health Organization Disability Assessment Schedule 2.0 [42]), which includes questions about difficulties in maintaining friendships in the last 30 days (Cronbach’s alpha for adjusted ‘getting along’ domain 0.9). Our division of social dysfunction (behavioral versus affective) is supported by higher inter-domain than cross-domain correlations between behavioral and affective social indicators (Table 2).

For patients, perceived social disability (WHO-DAS 2.0) was also assessed by the caregiver when available (most often parent or partner) and by the research staff conducting the assessment. Consistent with our earlier observations [43], correlations between the caregiver and researcher rated scores were high (*r* = .79 for SZ group; *r* = .79 for AD group, both p’s<0.001, S3I and S3II Table) and there were some missing values (not all patients participated in the study with a caregiver). In cases where both scores were available, we computed a ‘rater-perceived’ social disability score by calculating a mean score from both the caregiver and researcher rated score. In other cases, we used the available score (either caregiver or researcher rated). Correlations were strong for the SZ and HC groups between their self-rated perceived social disability score and the rater score (*r* = .84 for SZ; *r* = .90 for HC, p’s<0.001). For AD patients the correlation between the self-rated and rater-score was lower (r = .36). Corresponding to our earlier study on the self- and proxy-rated WHODAS scores [43], caregiver/researcher rated perceived social disability was higher than the rating by the AD patients themselves. We included the rater-perceived social disability in our analyses comparing patient groups.

All instruments are validated for both SZ and AD and were chosen after careful consideration [4].

### Severity of disease

Cognitive dysfunction was estimated in AD patients using the Alzheimer’s Disease Assessment Scale–Cognitive subscale (ADAS-cog) [44] which includes 13 tasks involving both subject-completed test and observer-based assessments such as word recall, naming objects, and orientation. Current states of positive and negative symptoms of schizophrenia were measured using the PANSS (positive and negative syndrome scale) [33].

### Mood characteristics

All participants (patients and controls) were asked to complete three mood questionnaires. The 16-item Quick inventory of Depressive Symptomatology (QIDS-SR) [36], the 20-item State-Trait Anxiety Inventory (STAI) [45] and the 20-item Positive and Negative Affect Scale (PANAS) [46] examined depressive symptoms, anxiety symptoms and current positive and negative affect states, respectively.

### Statistical analyses

Demographic and clinical characteristics were described using χ^2^ for dichotomous variables and t-tests for continuous variables. The Mann-Whitney test was used as nonparametric test when assumptions parametric testing were not met. Pearson correlations described associations between social functioning indicators and continuous demographics (age, years of education), point-biserial correlation coefficient described associations for binary demographics (sex, partner status and country). Analyses of covariance (ANCOVA’s) with post-hoc tests, all Bonferroni adjusted, compared social functioning indicators among SZ and AD patients, as well as their age-matched HC groups, while adjusting for age, sex, years of education, partner status and country. Effect sizes were calculated following Cohen’s formula for estimated differences [47]. To examine the association of disease severity and mood (the dependent variables) with behavioral and affective indicators of social functioning (independent variables), linear regression analyses were conducted, again while adjusting for age, sex, years of education, partner status and country. Statistical analyses were conducted using SPSS (IBM, version 24.0, IBM Corp., Armonk, NY, USA), and a two-tailed significance level of P < 0.05 was considered statistically significant.

## Results

HC participants had most years of education (17.2 and 16.7 for younger and older HC respectively), whilst both of the patient groups had on average 15.0 years of education (Table 1). SZ patients were less frequently with a partner (21.4%) as compared to younger HC (51.7%), AD patients (84.0%) and older HC (82.1%). As expected, psychotropic medication use was high in SZ patients (e.g. 89.3% used antipsychotics) and 43.8% of AD patients used acetylcholinesterase inhibitor and/or a NDMA receptor antagonist.

**Table 1:** Baseline characteristics (N = 163) across patient and control groups.

	Schizophrenia patients N = 56	Younger healthy controls N = 29	p-value	Alzheimer’s disease patients N = 50	Older healthy controls N = 28	p-value
**Demographics**						
Age, mean years (SD)	30.8 (6.4)	28.7 (7.4)	0.13	68.6 (7.2)	67.1 (7.0)	0.32
Sex (% female)	28.6%	41.4%	0.23	44.0%	46.4%	0.84
Education, mean years (SD)	15.0 (3.8)	17.2 (2.6)	0.001	15.0 (5.6)	16.7 (4.9)	0.21
Partner status (% with partner)	21.4%	51.7%	0.004	84.0%	82.1%	0.83
Country (% Spain)	39.1%	48.3%	0.43	42.0%	25.0%	0.13
**Specific disorder characteristics**						
Psychotropic medication						
Antipsychotic (%)	89.3%	0%		4.0%	0%	
Antidepressant (%)	19.6%	0%		16.0%	0%	
Acetylcholinesterase inhibitor or NDMA receptor antagonist (%)	0%	0%		43.8%	0%	
Benzodiazepines (%)	10.7%	0%		6.3%	7.1%	
Other psychotropics (%)	14.3%	0%		2.1%	3.6%	
Severity of disorder						
Schizophrenia severity						
Positive symptoms, mean PANSS (SD)	11.0 (3.4)	NA		NA	NA	
Negative symptoms, mean PANSS (SD)	14.6 (6.2)	NA		NA	NA	
AD severity, mean ADAS-Cog (SD)	NA	NA		26.9 (7.2)	NA	
**Mood characteristics**						
Depression severity, mean QIDS-SR (SD)	8.0 (5.3)	2.1 (1.5)	<0.001	4.1 (2.8)	2.0 (1.3)	<0.001
Anxiety severity, mean STAI (SD)	43.5 (11.2)	30.4 (5.9)	<0.001	30.7 (8.2)	27.5 (5.8)	0.08
Mood state, PANAS						
Positive Affect, mean (SD)	29.5 (6.6)	37.5 (5.9)	<0.001	32.5 (5.1)	38.2 (6.1)	<0.001
Negative Affect, mean (SD)	18.7 (6.5)	13.9 (3.0)	<0.001	13.9 (4.6)	12.7 (3.0)	0.24

Current positive and negative symptoms among SZ patients were 11.0 (SD ±3.4) and 14.6 (SD ±6.2) respectively on the PANSS (mean total score 51, SD ±13.1). Mean cognitive dysfunction of AD was 26.9 (SD ±7.2) on the ADAS-COG. SZ participants had more negative mood symptoms (i.e. more depressed mood, more anxiety, less positive affect and more negative affect) than all other groups (AD and both HC groups). Anxiety and negative affect were comparable between AD and their matched HC group, but AD patients had more depressive symptomatology and less positive affect (Table 1).

Correlation analyses (Table 2) across all participants show that age, educational level and partner status had significant associations with almost all social indices. However, sex and country had much less consistent associations with social indices (up to three associations were significant but without consistent direction of association).

**Table 2:** Pearson and point biserial correlations between social indicators and demographics in the overall sample (N = 163).

	Behavioral social indicators							Affective social indicators		
	Social withdrawal	Interpersonal functioning	Independence competence	Independence performance	Recreational activities	Prosocial activities	Total SFS score	Perceived social disability	Rater-perceived social disability	Loneliness
**Demographics**										
Age	.273***	.272**	-.162*	-.060	.376**	.119	.229**	-.305**	-.135	-.217**
Sex (female = 1; male = 0)	.160*	.036	-.009	.187*	.108	.028	.117	-.032	-.060	-.057
Education level	.148	.189*	.157*	.247**	.176*	.289**	.272**	-.071	-.261**	-.125
Partner status (yes = 1; no = 0)	.413**	.372**	.055	.012	.268**	.151	.332**	-.382**	-.342**	-.356**
Country (Spain = 1; Netherlands = 0)	-.189*	-.120	-.161*	-.039	-.140	.027	-.160*	-.013	.048	.201*
**Behavioral social indicators**										
Social withdrawal	1									
Interpersonal functioning	.541**	1								
Independence-competence	.256**	.320**	1							
Independence-performance	.283**	.361**	.605**	1						
Recreational activities	.437**	.389**	.359**	.440**	1					
Prosocial activities	.465**	.482**	.343**	.492**	.564**	1				
Total SFS score	.715**	.763**	.595**	.671**	.749**	.755**	1			
**Affective social indicators**										
Perceived social disability	-.572**	-.681**	-.357**	-.307**	-.414**	-.504**	-.662**	1		
Rater-perceived social disability	-.560**	-.598**	-.434**	-.483**	-.496**	-.616**	-.730**	.785**	1	
Loneliness	-.556**	-.615**	-.296**	-.278**	-.357**	-.393**	-.594**	.615**	.536**	1

*p-value < 0.05,

** p-value<0.01,

*** p-value<0.001.

### Comparison of patient groups with healthy controls

**Fig 1:**
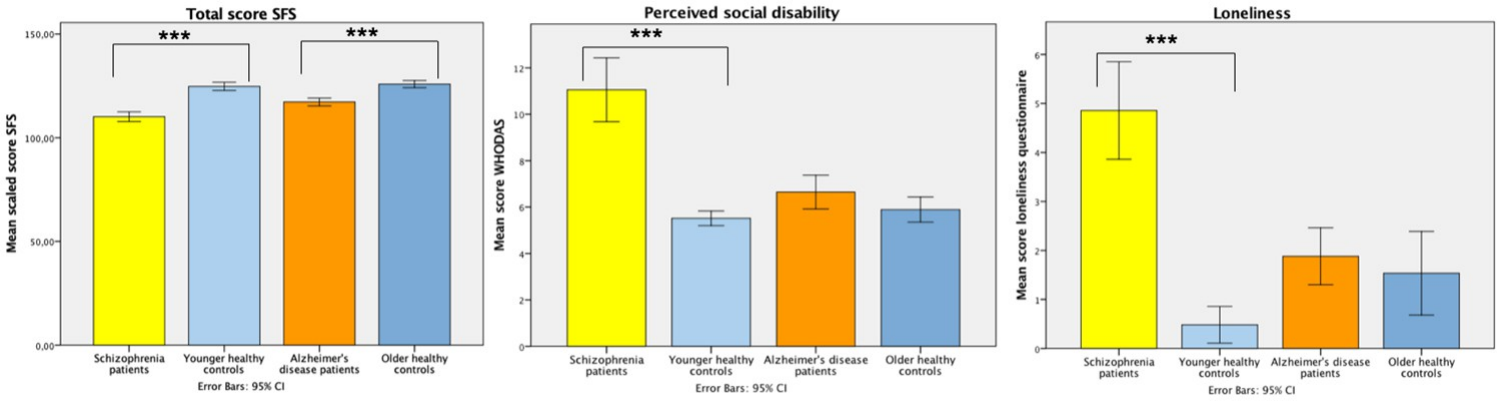
Unadjusted mean scores for total SFS, loneliness and perceived social disability across psychopathology. Error bars represent the standard error. Y axis depicts the different scores. *p-value < 0.05, ** p-value<0.01, *** p-value<0.001.

Fig 1 shows unadjusted means for the total SFS score as behavioral indicator, and self-rated perceived social disability and loneliness as affective social indicators per group with comparisons for SZ and AD with their age-matched controls.

It is clear in Table 3 that the least favorable social functioning outcomes on all measures were found for the SZ group with large effect sizes (Cohen’s *d’s* = 0.81–1.69) particularly pronounced for the affective indicators. AD patients showed less favorable outcomes for most behavioral social functioning indicators as compared to their matched HC group: independence-competence and -performance, recreational and prosocial activities and the total SFS score with medium to large effect sizes (*d’s* = 0.65–1.14). For both SZ and AD patients the total SFS was significantly lower from their age-matched controls with large effect sizes (d = 1.80 and 1.14 respectively). Affective social functional indicators of perceived social disability and loneliness, however, were not significantly different (*d’s* = 0.17 and *d* = 0.05 respectively) between AD and older HC participants. In contrast, the (mean score of caregiver and research staff) rater-perceived social disability was significantly worse in AD as compared to the older HC group (p<0.001, *d* = 1.29).

**Table 3:** Mean adjusted^†^ social functioning scores across disorders compared with healthy controls.

	Schizophrenia patients N = 56	Younger healthy controls N = 29	p-value	Effect size Cohen’s *d*	Alzheimer’s disease patients N = 50	Older healthy controls N = 28	p-value	Effect size Cohen’s *d*
**Behavioral social functioning indicators**								
Social withdrawal, mean (SE)	10.4 (0.5)	13.3 (0.6)	<0.001	1.30	12.3 (0.5)	13.1 (0.6)	0.74	0.35
Interpersonal functioning, mean (SE)	6.4 (0.4)	8.5 (0.4)	<0.001	1.33	8.1 (0.4)	8.8 (0.4)	0.43	0.66
Independence-competence, mean (SE)	36.2 (0.7)	38.7 (0.8)	0.002	0.81	33.9 (0.8)	37.0 (0.8)	<0.001	1.06
Independence-performance, mean (SE)	29.4 (1.1)	34.6 (1.3)	<0.001	1.04	28.1 (1.2)	32.4 (1.3)	<0.001	0.96
Recreation activities, mean (SE)	19.3 (1.4)	24.5 (1.7)	0.002	0.81	23.2 (1.6)	28.3 (1.7)	0.002	0.83
Prosocial activities, mean (SE)	24.8 (2.1)	36.8 (2.6)	<0.001	1.25	28.6 (2.4)	34.5 (2.5)	0.030	0.65
Total SFS score, mean (SE)	111.2 (1.7)	125.3 (2.1)	<0.001	1.80	116.5 (1.9)	124.9 (2.0)	<0.001	1.14
**Affective social functioning indicators**								
Perceived social disability, mean (SE)	11.3 (0.8)	6.1 (1.0)	<0.001	1.39	6.1 (0.9)	5.5 (1.0)	1.00	0.17
Rater-perceived social disability, mean (SE)	12.2 (0.8)	5.7 (1.0)	<0.001	1.69	10.5 (1.0)	6.0 (1.0)	<0.001	1.29
Loneliness, mean (SE)	4.6 (0.6)	0.4 (0.8)	<0.001	1.45	2.0 (0.7)	1.8 (0.8)	1.00	0.05

^†^ Adjusted for age, sex, years of education, partner status and country. Bonferroni adjustment for multiple comparisons.

### Comparisons across/within patient groups

In the cross-disorder comparison between SZ and AD patients, (Table 4), all behavioral social indicators were comparable, as well as the total SFS score (p = 0.70, *d* = 0.31). Perceived social disability showed a significantly poorer outcome for SZ patients as compared to AD participants (Cohen’s *d* = 0.65, p = 0.008). The self-reported perceived social disability was higher in SZ compared to AD patients, but was not significantly different for the rater-perceived social disability.

**Table 4:** Mean adjusted^†^ social functioning scores across disorders with effect sizes (N = 106).

	Schizophrenia patients N = 56	Alzheimer’s disease patients N = 50	p-value	Effect size *d*
**Behavioral social indicators**				
Social withdrawal, mean (SE)	10.4 (0.5)	12.3 (0.5)	0.27	0.40
Interpersonal functioning, mean (SE)	6.4 (0.4)	8.1 (0.4)	0.08	0.28
Independence-competence, mean (SE)	36.2 (0.7)	33.9 (0.8)	0.54	0.33
Independence-performance, mean (SE)	29.4 (1.1)	28.1 (1.2)	1.00	0.13
Recreational activities, mean (SE)	19.3 (1.4)	23.2 (1.6)	0.98	0.27
Pro-social activities, mean (SE)	24.8 (2.1)	28.6 (2.4)	1.00	0.18
Total SFS score, mean (SE)	111.2 (1.7)	116.5 (1.9)	0.70	0.31
**Affective social indicators**				
Perceived social disability, mean (SE)	11.3 (0.8)	6.1 (0.9)	0.008	0.65
Rater-perceived social disability, mean (SE)	12.2 (0.8)	10.5 (1.0)	1.00	0.20
Loneliness, mean (SE)	4.6 (0.6)	2.0 (0.7)	0.21	0.41

^†^ Adjusted for age, sex, years of education, partner status and country. Bonferroni adjustment for multiple comparisons.

Linear regression analyses (Table 5) examined the association between clinical characteristics (severity of disorder, mood, positive/negative affect) and social functioning indices among SZ and AD patients adjusted for aforementioned covariates. Overall, only a few predictors were found to associate strongly with overall disease severity. More AD symptomatology was associated with fewer prosocial activities (β = -0.400, p = 0.014). More negative SZ symptomatology was associated with less interpersonal functioning (β = -0.330, p = 0.010), and more positive SZ symptoms related to more loneliness (β = 0.278, p = 0.049). Disease severity was not associated with the total SFS.

**Table 5:** Adjusted^†^ associations of clinical characteristics and various social functioning indicators within the group of patients (AD N = 50, SZ = 56).

	ADAS-Cog AD only β	PANSS positive symptoms SZ only β	PANSS negative symptoms SZ only β	Depression severity AD and SZ β	Anxiety severity AD and SZ β	PANAS positive affect AD and SZ β	PANAS negative affect AD and SZ β
**Behavioral social indicators**							
Social withdrawal	0.173	-0.224	-0.053	-0.555***	-0.413***	0.414***	-0.355**
Interpersonal functioning	-0.059	-0.072	-0.330*	-0.522***	-0.424***	0.468***	-0.309**
Independence-competence	-0.166	0.009	-0.224	-0.151	-0.132	0.158	-0.175
Independence-performance	-0.158	-0.039	-0.151	-0.102	-0.121	0.166	-0.091
Recreational activities	-0.263	-0.124	-0.021	-0.166	-0.169	0.395***	-0.158
Pro-social activities	-0.400*	-0.054	-0.077	-0.312**	-0.165	0.366***	-0.043
Total SFS score	-0.242	-0.111	-0.205	-0.498***	-0.399***	0.561***	-0.310**
**Affective social indicators**							
Perceived social disability	0.194	-0.082	0.238	0.650***	0.423***	-0.516***	0.274**
Rater-perceived social disability	0.322	-0.274	0.177	0.424***	0.232*	-0.336**	0.125
Loneliness	-0.163	0.278*	0.227	0.653***	0.495***	-0.370**	0.445***

^†^ adjusted for sex, age, educational years, partner status and country.

*p-value < 0.05,

** p-value<0.01,

*** p-value<0.001.

More consistent associations were found for mood state indicators (Table 5). Across both diagnostic groups, positive affect was associated with all behavioral and affective social functioning indicators, except for independence-competence and -performance. More favorable social functioning was found for those with less depressive symptomatology, less anxiety and better mood state. Across patient groups, positive mood and lower depression and anxiety levels were thus strongly associated with better social functioning, even more so than severity of disease per patient group.

## Discussion

The current study presents novel findings from the pan-European PRISM project on cross-disorder and disorder-specific deficits in social functioning among SZ and AD patients. As compared to HC, both behavioral and affective social functioning are clearly poorer in SZ patients (Cohens *d’s* 0.81–1.69), whereas AD patients have mostly poorer behavioral social function (Cohen’s *d’s* 0.65–1.14). Behavioral indices of social functioning were fairly similar across patient groups, SZ patients have more feelings socially disability than AD patients (Cohen’s *d* 0.65). Across patient groups, positive mood, lower depression and anxiety levels were strong determinants of better social functioning (p’s <0.001), even more so than severity of disease. Overall, this indicates that SZ and AD patients have rather similar social functioning levels in terms of behavior, but affective social functioning is different.

Consistent with our earlier observations [43], we observed a putative lack of insight among AD patients, who perceive their social disability on the same level as healthy controls, whereas AD informants and research staff perceive their social disability as significantly more impaired. By contrast the current data suggest that SZ patients are more aware of their own social disabilities than AD patients, an observation that warrants further investigation in future studies [48]. Findings are in line with previous research in AD patients where it was shown that a strong self-concept was associated with larger social dysfunction discrepancy comparing self-rating with caregiver rating [24]. Interestingly, social functioning in both diagnostic groups was found to be more strongly associated with mood than by current state of the disorders.

For SZ patients impairments in behavioral social functioning are consistently described [14, 49, 50], as are impairments in affective social functioning, such as feeling lonely and socially impaired [51–53]. Our results seem consistent with and replicate this previous SZ research. Findings for AD patients are less unequivocal. Impairments in behavioral social functioning for AD patients are described as rather subtle in an early stage of the disease [21, 23]. In our study, however, AD patients differed from their matched HC group on most behavioral social functioning indices. It is possible that broad assessment of social functioning as implemented here could easier reveal subtle differences in specific aspects of social behavior. Among affective social indicators, feelings of loneliness were similar between AD and their matched healthy controls, as was found before [54].

When comparing SZ and AD patients on behavioral social functioning, a highly similar pattern of (dys)functioning emerged. This was especially true for the combined SFS outcomes. The largest differences between the patient groups emerged for the affective social indicators. For instance, patients with SZ evaluated themselves as less capable in social contact than AD patients. This suggests that patients have quite similar social functioning behavior but may evaluate this differently. It has been shown that feelings of loneliness are not necessarily reflected by objective social isolation [51, 55]. AD patients evaluated their perceived social disability on the same level as their matched healthy controls, whereas an informant evaluated their social abilities as strongly impaired. This suggest that AD patients may have an overly positive view of their own social functioning, possibly leading to underreporting of their impairments. This overestimation of their own social functioning, as compared to informants, has been described before [24, 43] and has been linked to loss of awareness due to AD [23]. Lack of awareness of social dysfunction is related to greater distress among the caregivers of AD patients [20, 23]. Mood influenced social functioning to a large extent, especially the affective indicators, with better mood associated with more favorable affective social outcomes. The large impact of anxiety and depressive disorders on social functioning has been described before, although the underlying pathophysiological mechanisms remain largely unknown. One hypothesis argues that imbalanced brain network function (especially in the brain’s default mode network [56]) not only impedes adaptive social functioning, but also interferes with maintenance of stable (positive) mood [9, 57, 58]. Current severity of disease in SZ and AD had only minor influence on social functioning indicators, as was previously found for AD [22] and SZ [49], although the reverse has been described as well [50, 54].

The PRISM study described here is unique in its comparison of two patient groups with distinctive pathologies and age-ranges, in relation to various indicators of social functioning. Transdiagnostic research is limited by the fact that similar, uniform instruments are often not used in different disease populations. We argue that such standardization of specific outcomes is necessary to progress the transdiagnostic research field. It could be that underlying contributing factors to social functioning are not perse similar across different disease conditions, but this does not mean that certain outcomes, like social functioning, cannot be measured in a uniform way. Some limitations inherent to the study should be mentioned. First, our data are cross-sectional, thereby not allowing for causal interpretations. Second, data is based on self- or informer-based evaluation, which may have resulted in biased responding. Passive monitoring of social behavior is a promising development to objectively measure ambulatory behavior and tackle possible self-report bias. Smartphone technology such as the BEHAPP-app [43, 59, 60] will allow the comparison of perceived social functioning with objective measurements. Third, we excluded the employment subscale of the SFS, since most AD patients and older HC’s were retired or not working, in line with other research [38]. However, inclusion of the employment subscale would arguably increase differences in social functioning between younger SZ patients and younger HC’s. Fourth, we made a distinction between behavioral (i.e. more quantitative) and affective (i.e. more perceptions of own social dysfunction) social indicators, which bears some clear benefits and is supported by prior work, but may ultimately pose an oversimplification of a complex phenomenon. Future work is therefore warranted to further explore and validate the findings and interpretations of the current study.

In summary, the present study reveals that behavioral aspects of social functioning are rather similarly affected in SZ and AD, whilst the affective perception of this behavior seems partly different in the two patient groups. Patients diagnosed with AD evaluate their interpersonal relations as only mildly impaired, whereas patients diagnosed with SZ have a more negative perspective of their own social functioning. AD patients may underreport their impairments in social functioning. Future research on social functioning should make a distinction between behavioral and affective indicators of social functioning, as these may associate differentially with pathophysiology. Findings further suggest that mood is highly associated with social functioning. This study may serve as a point of departure for future cross-disorder studies into the neurobiological underpinnings of social impairments in SZ and AD.

## Supporting information

S1 Table Factor analyses of the six subscales of the social functioning scale. (DOCX)

S2 Table Mean scaled social functioning scale scores (N = 163). (DOCX)

S3 Table I. Perceived social disability (WHO-DAS score) availability across patient groups. II. Perceived social disability correlations across groups.
(DOCX)
