# Supplementary material for: Cross-disorder and disorder-specific deficits in social functioning among schizophrenia and alzheimer’s disease patients
Source: PLoS One. 2022 Apr 14;17(4):e0263769. doi: 10.1371/journal.pone.0263769 (PMC9009658; doi:10.1371/journal.pone.0263769)
Supplement: S1 Table — (DOCX) [file pone.0263769.s001.docx]

**Supplement 1**

**S1 Table 1. Factor analyses of the six subscales of the Social Functioning Scale**

|  | **Factor loading**  (N=164) |
| --- | --- |
| Withdrawal | .685 |
| Interpersonal | .714 |
| Independence-competence | .653 |
| Independence-performance | .736 |
| Recreation | .745 |
| Prosocial | .787 |
| Eigenvalue | 3.121 |
| % Variance explained | 52.02 |
