# Supplementary material for: Cross-disorder and disorder-specific deficits in social functioning among schizophrenia and alzheimer’s disease patients
Source: PLoS One. 2022 Apr 14;17(4):e0263769. doi: 10.1371/journal.pone.0263769 (PMC9009658; doi:10.1371/journal.pone.0263769)
Supplement: S2 Table — (DOCX) [file pone.0263769.s002.docx]

**Supplement 2**

**S2 Table 1. Mean scaled social functioning scale scores (N=163)**

|  | **Schizophrenia patients**  N = 56 | **Younger healthy controls**  N = 29 | **Alzheimer’s disease patients**  N=50 | **Older healthy controls**  N= 28 |
| --- | --- | --- | --- | --- |
| Social withdrawal, mean (SD) | 102.7 (11.2) | 120.5 (10.9) | 114.4 (10.4) | 120.8 (10.6) |
| Interpersonal functioning, mean (SD) | 112.8 (19.0) | 140.0 (10.5) | 130.1 (17.1) | 140.8 (9.5) |
| Independence-competence, mean (SD) | 110.9 (9.8) | 120.0 (5.6) | 106.4 (8.4) | 117.1 (6.7) |
| Independence-performance, mean (SD) | 108.3 (10.0) | 118.3 (7.6) | 107.0 (8.4) | 115.5 (6.0) |
| Recreation activities, mean (SD) | 107.0 (8.4) | 120.2 (13.5) | 120.4 (14.8) | 132.4 (10.5) |
| Prosocial activities, mean (SD) | 118.6 (12.4) | 131.8 (6.4) | 124.5 (9.2) | 130.9 (8.9) |
| Total SFS score, mean (SD) | 111.2 (1.7) | 125.3 (2.1) | 116.5 (1.9) | 124.9 (2.0) |
