# Supplementary material for: Cross-disorder and disorder-specific deficits in social functioning among schizophrenia and alzheimer’s disease patients
Source: PLoS One. 2022 Apr 14;17(4):e0263769. doi: 10.1371/journal.pone.0263769 (PMC9009658; doi:10.1371/journal.pone.0263769)
Supplement: S3 Table — I. Perceived social disability (WHO-DAS score) availability across patient groups. II. Perceived social disability correlations across groups. (DOCX) [file pone.0263769.s003.docx]

**Supplement 3**

**S3. Table I. Perceived social disability (WHO-DAS score) availability across patient groups**

|  | **Schizophrenia patients**  **(N=56)** | **Alzheimer’s disease patients**  **(N=50)** |
| --- | --- | --- |
| Patient rated score, mean (SD) | 11.1 (5.1) 0 missing | 6.4 (2.1) 0 missing |
| Caregiver rated score, mean (SD) | 12.4 (4.5) 31 missing | 9.8 (3.4) 12 missing |
| Researcher rated score, mean (SD) | 12.7 (4.8) 7 missing | 10.8 (3.8) 11 missing |

**S3. Table II. Perceived social disability correlations across groups**

|  | **Participant rated**  Total (n=163) | **Caregiver rated**  Total | **Participant rated**  SZ (N=56) | **Caregiver rated**  SZ | **Participant rated**  AD (N=50) | **Caregiver rated**  AD | **Participant rated**  HC (N=57) |
| --- | --- | --- | --- | --- | --- | --- | --- |
| **Caregiver rated** | .47** | 1 | .60** | 1 | .29* | 1 | NA |
| **Researcher rated** | .71** | .80** | .83** | .79** | .25 | .79** | .84** |

*p-value < 0.05 ** p-value<0.001

Abbreviations: SZ; schizophrenia, AD: Alzheimer’s disease, HC: healthy controls.
